# Multi-omics analysis reveals the molecular basis of flavonoid accumulation in fructus of Gardenia (*Gardenia jasminoides* Ellis)

**DOI:** 10.1186/s12864-023-09666-x

**Published:** 2023-10-04

**Authors:** Jianrong Chen, Weizhuo Tang, Chunyan Li, Ding Kuang, Xiaojiang Xu, Yuan Gong, Fang Liu, Song Gao

**Affiliations:** 1 College of Biological and Chemical Engineering, Changsha University, Changsha, China; 2 Hunan Yangli Agriculture and Forestry Sci-Tech Co., Ltd, Yueyang, China; 3 College of Horticulture and Landscape Architecture, Yangzhou University, Yangzhou, China

**Keywords:** Gardenia (*Gardenia jasminoides* Ellis), Flavonoid metabolism, Geniposide biosynthesis, Multi-omics

## Abstract

**Background:**

The fruits of Gardenia are rich in flavonoids and geniposides, which have various pharmacological effects such as antioxidant, anti-inflammatory and anticancer. In this study, we analyzed the transcriptome and metabolome of gardenia peel and kernel at different growth stages, revealed the regulatory network related to flavonoid synthesis, and identified the key regulatory genes.

**Results:**

The results showed that in terms of flavonoid metabolic pathways, gardenia fruits mainly synthesized cinnamic acid through the phenylpropanoid pathway, and then synthesized flavonoids through the action of catalytic enzymes such as 4-coumaroyl-CoA ligase, chalcone synthase, chalcone isomerase and flavanol synthase, respectively. In addition, we found that the metabolomics data showed a certain spatial and temporal pattern in the expression of genes related to the flavonoid metabolism pathway and the relative content of metabolites, which was related to the development and ripening process of the fruit.

**Conclusions:**

In summary, this study successfully screened out the key genes related to the biosynthesis metabolism of flavonoids in gardenia through the joint analysis of transcriptome and metabolome. This is of certain significance to the in-depth study of the formation mechanism of gardenia efficacy components and the improvement of quality.

**Supplementary Information:**

The online version contains supplementary material available at 10.1186/s12864-023-09666-x.

## Background

Gardenia (*Gardenia jasminoides* Ellis, Zhizi in Chinese) is a shrub plant of the genus Gardenia in the Rubiaceae family. The Chinese species of gardenia has been cultivated for at least 1,000 years and was introduced to Europe and the America in the mid-18th century. Its fruit mainly contains cyclic enol ether terpenes, pigments, flavonoids, organic acids, and other active ingredients [1, 2], with anti-inflammatory, antioxidant, choleretic and diuretic, anti-tumor, antipyretic, analgesic, hypolipidemic and many other pharmacological activities, has long been used as food and traditional Chinese medicine [3–9]. Gardenia fruit is rich in flavonoids, the main components are gardenia yellow pigment, isogardenia yellow pigment, quercetin and so on [2].

Flavonoids are widely found in plants in nature and are secondary plant metabolites, with over 6000 individual compounds know, include chalcones, dihydroflavonoids, flavonoids, and anthocyanins [10]. The physiological and pharmacological activities of flavonoids have been studied extensively in recent years, such as antioxidant, anti-aging, anti-bacterial, anti-tumor and anti-viral [11, 12]. Iridoid glycosides (geniposide, gardenoside, and gardoside) are the active constituents of mature gardenia fruits [13]. Among them, geniposide have a variety of uses, different conditions of fermentation, could be made into natural edible coloring agent gardenia blue and gardenia red [14], but also for the treatment of cardiovascular, hepatobiliary, and other diseases and diabetes raw material drugs [15]. In vitro proliferation and differentiation of neural stem cells were also promoted [16].

It is of great significance to study the key genes of flavonoid metabolic pathway and geniposide biosynthesis in the fruits of gardenia to have an in-depth understanding of the mechanism of the formation of gardenia’s effective components. At present, the specific composition and content of flavonoids and geniposide substances in gardenia fruits are still unclear, and the key regulatory genes are also unclear. Therefore, this study intends to take gardenia fruits (peel and kernel) at different growth stages as the research object and carry out systematic analysis by using metabolome combined with transcriptome to clarify the relative content of each component of flavonoids and geniposide substances and the expression difference of key regulatory genes, identification of key genes and metabolic pathways involved in the biosynthesis of bioactive compounds in gardenia fruits. This provides a theoretical basis for the in-depth research on the formation mechanism of gardenia efficacy components and improving the quality of gardenia fruit.

## Results

### Phenotypes of gardenia fruits at different growth stages differences and metabolome overview

**Fig. 1:**
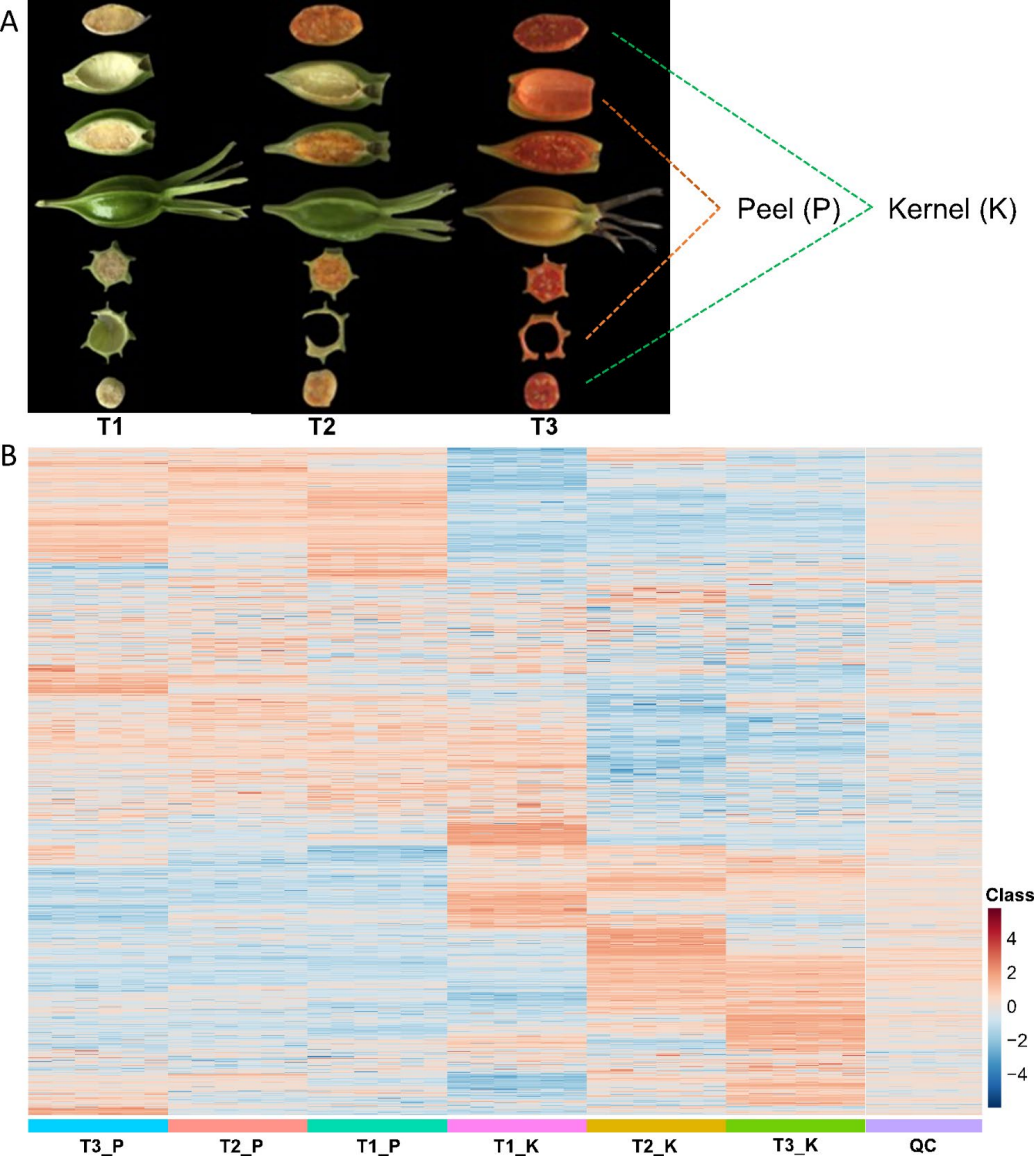
**Profile of two tissues and metabolites of gardenia fruit at different growth stages**. (**A**), Different tissues of gardenia fruits at different growth stages. (**B**), Heatmap analysis of metabolites of different samples in POS mode. The shades of color indicate more or fewer metabolites, with red representing more and blue representing less. T1 represent green ripe fruits (sampled on July 16), T2 represent semi-ripe fruits (sampled on August 15) and T3 represent yellow ripe fruits (sampled on October 20). Peel (P) and Kernel (K). The heatmap of the NEG mode was shown in the supplemental Fig. S5

Known for its medicinal properties, gardenia fruits were commonly used in traditional Chinese medicine. The phenotype of gardenia fruit was greatly at different stages of maturation. The color of gardenia fruit changes from green to yellow as it matures. The size and shape of the fruit varies at different stages of ripening, with the immature fruit being oval and smaller in size, while the ripe fruit is larger (Fig. 1A). To investigate the changes of metabolites during the development of gardenia fruits, we performed metabolomics analysis. The metabolites of gardenia fruit were analyzed and determined based on UPLC-MS/MS. The final statistics showed that the POS (positive) and NEG (negative) modes yielded 23,000 and 20,090 candidate metabolites, respectively, in the HMDB-level 1 identification results and all of them were attributed to the HMDB database, of which 9,935 and 10,933 metabolites were included in “Lipids and lipid-like molecules”, and 4,969 and 5,078 metabolites were included in “Phenylpropanoids and polyketides” (Table S1, Fig. S1). In the POS and NEG modes, 5481 and 3439 metabolites were classified into 40 and 38 KEGG pathway level 2, of which 2391 and 925 metabolites were classified as “metabolism”, of which 43 and 42 were classified as “Metabolism of terpenoids and polyketides” (Fig. S2). The number of metabolites involved in “Phenylalanine metabolism” in the POS and NEG modes were 10 and 8, respectively (Fig. S3).

The signal intensity information of each substance in different samples was extracted using XCMS software and quality controlled using metaX software: firstly, low-quality peaks were removed, followed by missing value filling using KNN (K-Nearest Neighbors) method, followed by PQN (Probabilistic Quotient Normalization) and QC-RSC (QC-robust spline batch correction) were used for data normalization. High-quality metabolites were quantified to 6667 and 6086 in the POS and NEG modes, respectively (Fig. 1B, Table S2).

### Metabolic differences in different tissues of Gardenia fruits at different growth stages

**Fig. 2:**
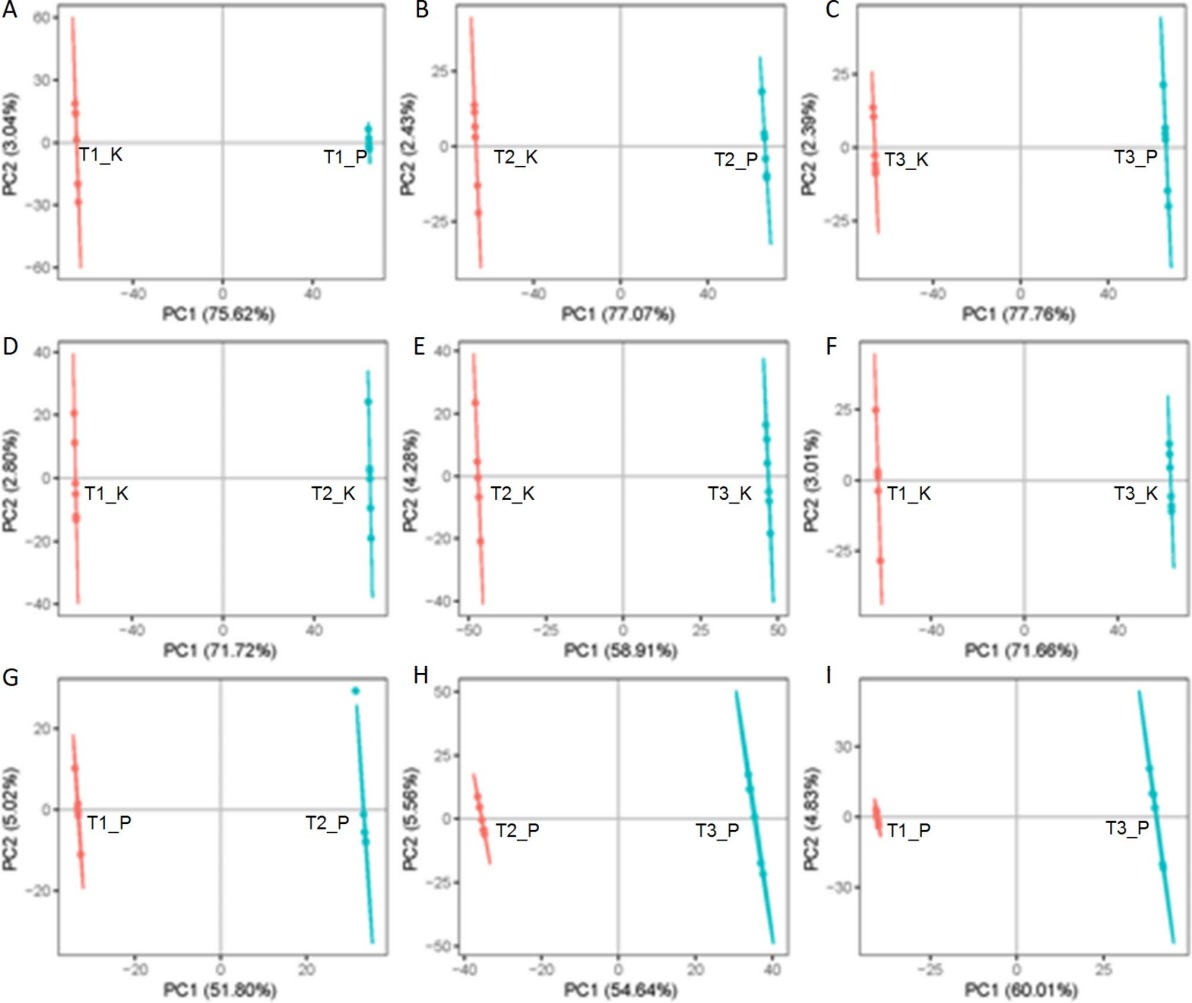
**The OPLS-DA score plots for different comparison groups**. (**A**-**I**), T1_K vs. T1_P, T2_K vs. T2_P, T3_K vs. T3_P, T1_K vs. T2_K, T2_K vs. T3_K, T1_K vs. T3_K, T1_P vs. T2_P, T2_P vs. T3_P, T1_P vs. T3_P. T1 represent green ripe fruits (sampled on July 16), T2 represent semi-ripe fruits (sampled on August 15) and T3 represent yellow ripe fruits (sampled on October 20). Peel (P), kernel (K)

We used the OPLS-DA model to reveal the differences between different growth stages of gardenia fruits and between different tissues (kernel and peel), with the horizontal coordinates indicating the differences between samples of the same group and the vertical coordinates indicating the differences between six replicates in each sample (Fig. 2A-I), and the six replicates in each sample were clustered together in the PCA analysis, while the different samples were clearly separated, showing the high reproducibility and reliability of the data in this study (Fig. S4). Using univariate analysis of variance multiplicity and t.test statistical tests, BH correction was performed to obtain q-value, and the Variable Important for the Projection (VIP) values obtained by multivariate statistical analysis PLS-DA were combined to screen for differentially expressed metabolites (DEMs). DEMs were screened for each group of samples based on fold change ≥ 2 or fold change ≤ 0.5 and VIP ≥ 1. There were 769 and 1229 metabolites that showed upregulation and downregulation, respectively, in comparisons of T1_K vs. T1_P (Table S2).

**Fig. 3:**
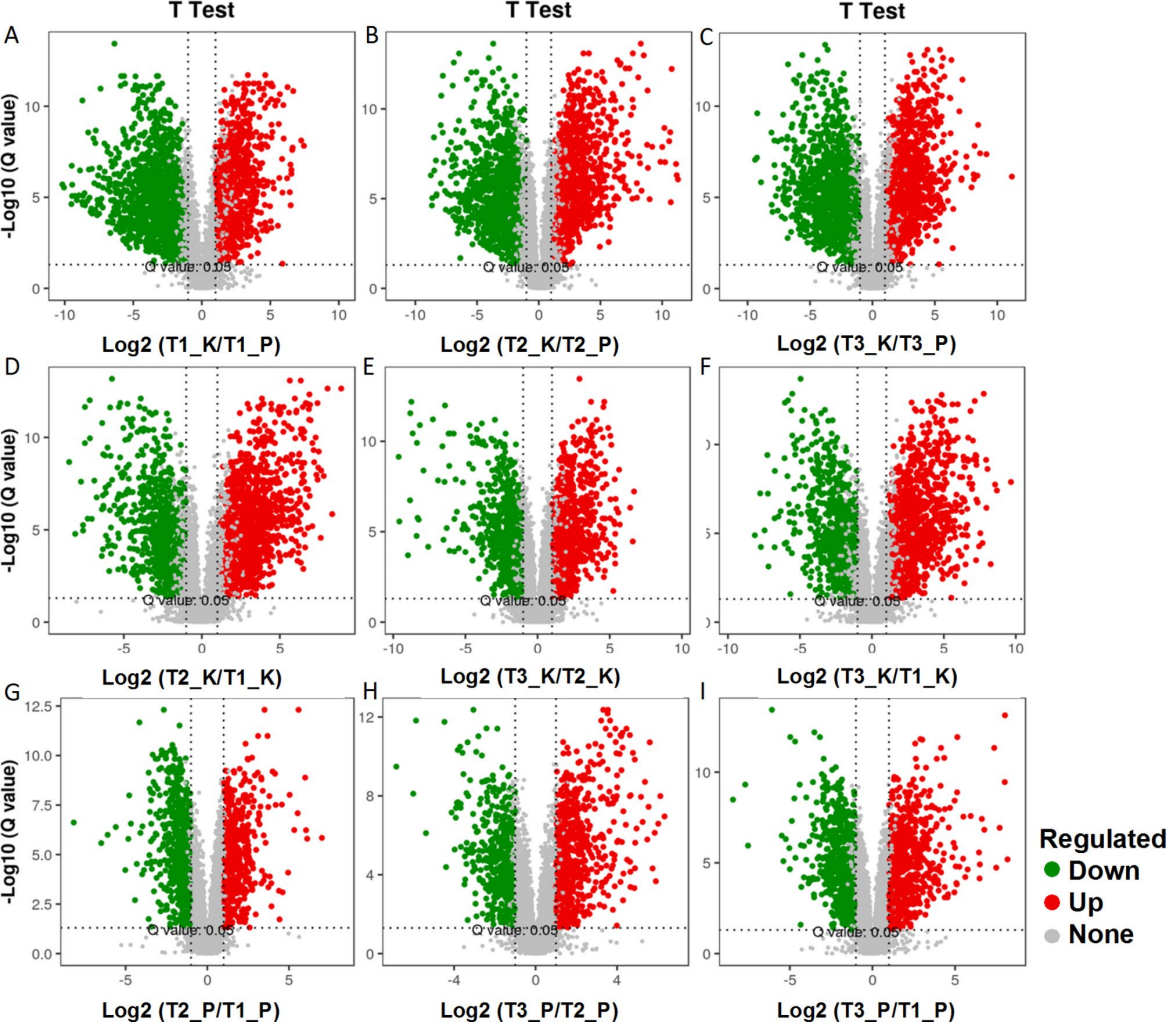
**Volcano plot analysis of differential metabolites in different tissue comparison groups of gardenia fruits at different maturity stages**. (**A**-**I**), T1_K vs. T1_P, T2_K vs. T2_P, T3_K vs. T3_P, T2_K vs. T1_K, T3_K vs. T2_K, T3_K vs. T1_K, T2_P vs. T1_P, T3_P vs. T2_P, T3_P vs. T1_P. Red dots are upregulated metabolites, blue dots are downregulated metabolites. T1 represent green ripe fruits (sampled on July 16), T2 represent semi-ripe fruits (sampled on August 15) and T3 represent yellow ripe fruits (sampled on October 20). Peel (P), kernel (K)

In addition, 40 flavonoids and 11 geniposides were identified among these metabolites, of which 11 flavonoid metabolites and 7 geniposides metabolites were significantly different (Table S3). It was found that the accumulation of these metabolites varied between different tissues at the same growth stage and between the same tissues at different growth stages. We identified a total of 4684 DEMs in the 9 comparison groups (Fig. 3A-I, Table S4), including 1998, 2033 and 2146 in T1_K vs. T1_P, T2_K vs. T2_P, T3_K vs. T3_P, respectively; 1898, 1467 and 1853 in T2_K vs. T1_K, T3_K vs. T2_K, T3_K vs. T1_K, respectively; 1144, 1164 and 1365 in T2_P vs. T1_P, T3_P vs. T2_P, T3_P vs. T1_P, respectively (Table S4).

By comparing the relative contents of metabolites at different growth stages, we found that the relative content of geniposide in the kernel of gardenia was significantly lower than that in the peel of the fruit, suggesting that geniposide were mainly present in the peel of gardenia. Surprisingly, the relative content of the flavonoids, such as “Artocarpin”, did not differ significantly between the kernels and the peel during the three growth stages, whereas the relative content of “Atalantoflavone” increased significantly in the peel with the growth of gardenia (Table S3). Further analysis revealed that the relative content of geniposide in the peel of gardenia at the second growth stage showed a decreasing trend, and with the growth of the fruit, the content rose again, but did not rise to the content in the early growth stage, which is a strange phenomenon. The relative content of “Atalantoflavone” was significantly higher in T2 compared to T1, but there was no difference between T3 and T2 (Table S5).

### Transcriptome sequencing reveals genes differentially expressed in different tissues of gardenia fruits at different growth stages

**Fig. 4:**
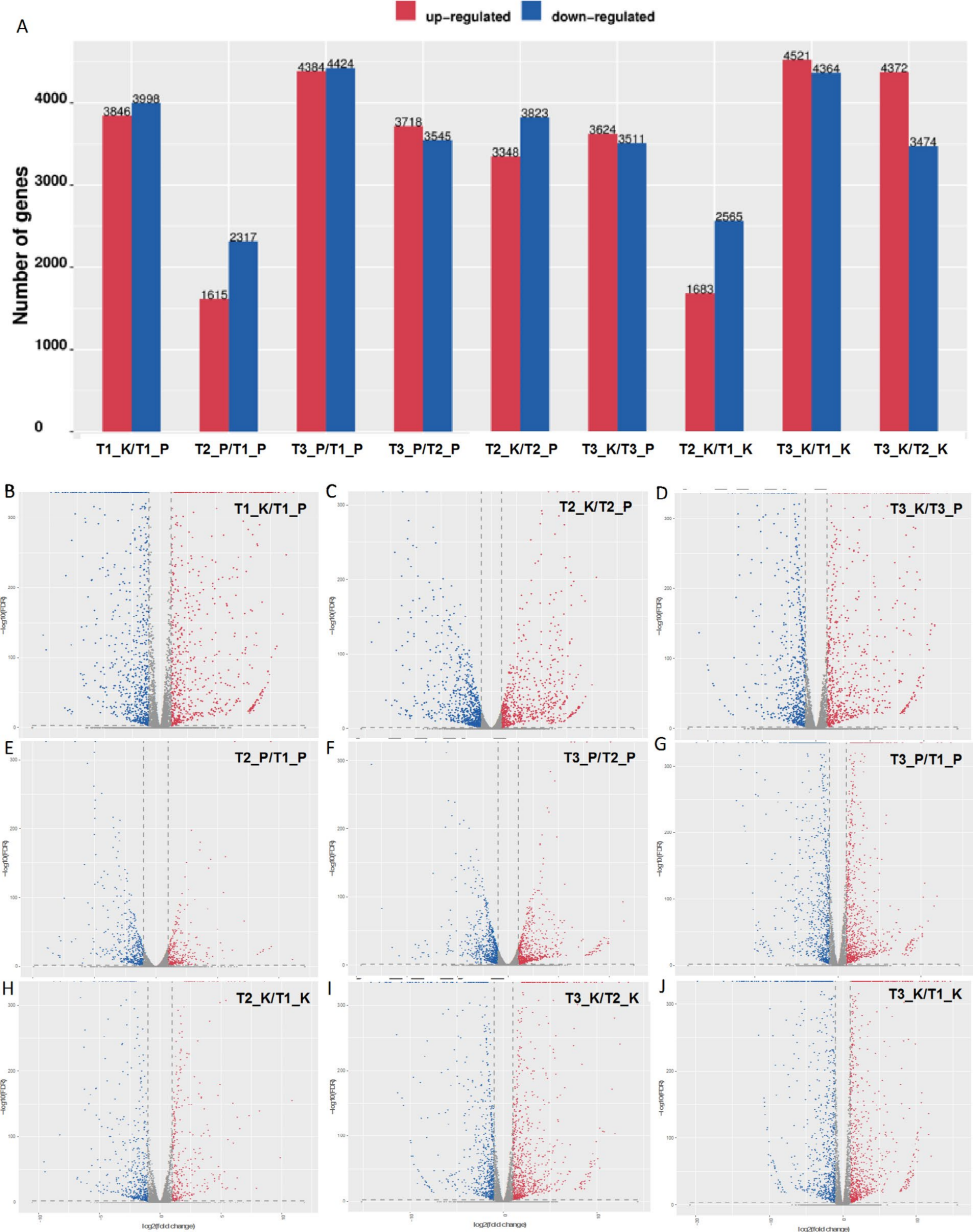
**Differentially expressed genes in different tissue comparison groups of gardenia fruits at different maturity stages**. (**A**), Comparison of the number of differential genes in different groups. (**B**-**J**), Volcano plot analysis of T1_K vs. T1_P, T2_K vs. T2_P, T3_K vs. T3_P, T2_K vs. T1_K, T3_K vs. T2_K, T3_K vs. T1_K, T2_P vs. T1_P, T3_P vs. T2_P, T3_P vs. T1_P. Red dots are upregulated metabolites, blue dots are downregulated metabolites. T1 represent green ripe fruits (sampled on July 16), T2 represent semi-ripe fruits (sampled on August 15) and T3 represent yellow ripe fruits (sampled on October 20). Peel (P), kernel (K)

A total of 31,660 genes were identified and annotated by transcriptome data analysis, and a total of 17,078 differentially expressed genes (DEGs) were obtained from 9 comparison groups by differential analysis, with DEGs between samples as shown in Fig. S5. In the volcano plots, the levels of DEGs were also statistically significant among different tissues of gardenia fruits at different stages (Fig. 4B-J). Interestingly, DEGs appeared to be different in different groups. For example, by comparing the number of DEGs between groups, we found that T3_P vs. T1_P and T3_K vs. T1_K had the highest number of DEGs, and T2_P vs. T1_P and T2_K vs. T1_K had the lowest number of DEGs (Fig. 4A). These results suggested that maturity of gardenia fruit plays an important role in gene expression.

### Analysis of DEGs in the flavonoid synthesis pathway

**Fig. 5:**
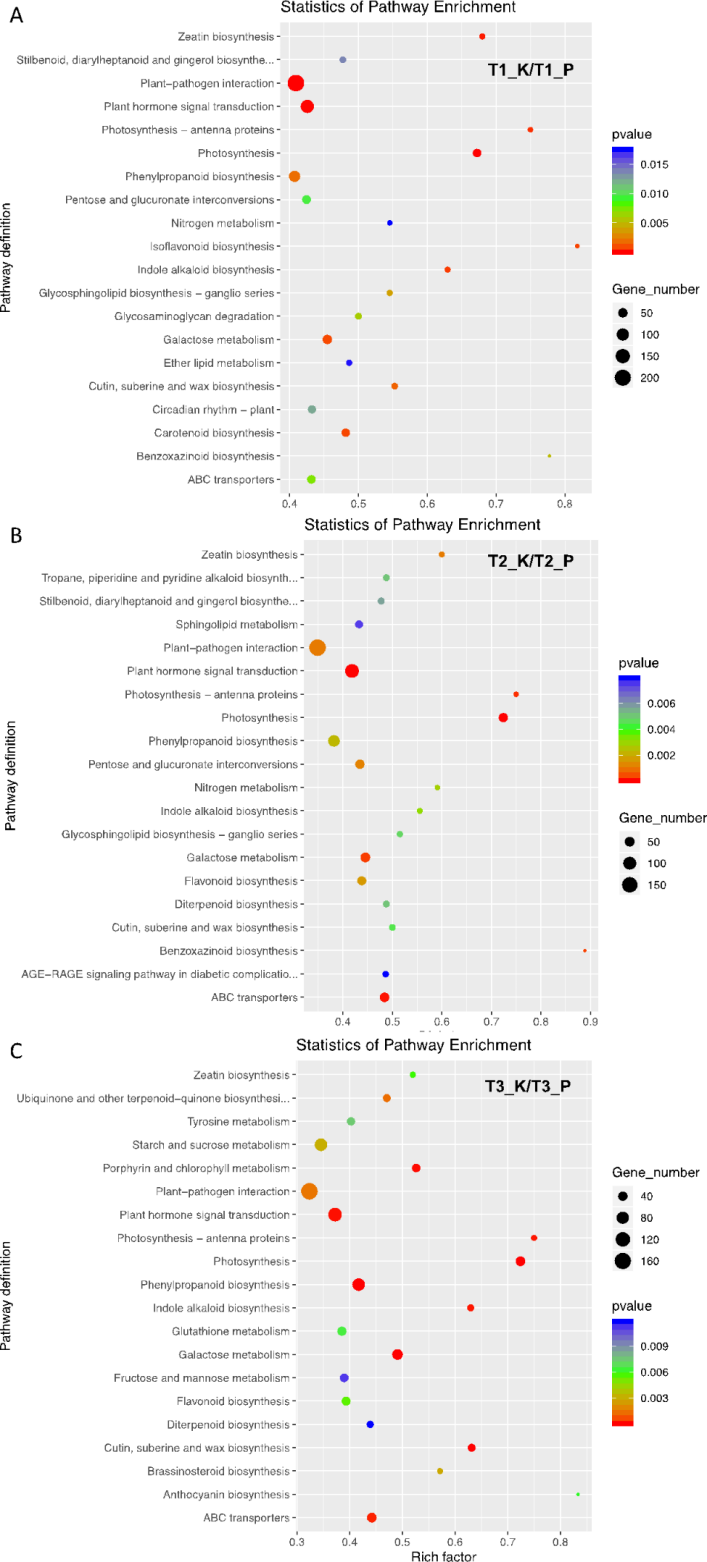
**KEGG enrichment analysis of differentially expressed genes among different tissues of Gardenia fruits at different growth stages**. (**A**-**C**), T1_K vs. T1_P, T2_K vs. T2_P, T3_K vs. T3_P. Horizontal coordinates represent the enrichment factor, vertical coordinates represent the pathway definition, dot size represents the number of genes, and color represents the *p*-value. T1 represent green ripe fruits (sampled on July 16), T2 represent semi-ripe fruits (sampled on August 15) and T3 represent yellow ripe fruits (sampled on October 20). Peel (P), kernel (K)

Flavonoids and iridoids compounds (geniposides) are the main active components of gardenia fruits [17]. Therefore, we focused on DEGs involved in flavonoid biosynthesis. KEGG enrichment analysis of DEGs showed that 75 genes were enriched in the flavonoid biosynthesis pathway (Table S6). KEGG analysis also revealed 47 and 40 DEGs associated with flavonoid synthesis in T1_K vs. T1_P, T2_K vs. T2_P and T3_K vs. T3_P, respectively. We mapped these key genes to the reference genome of gardenia, and these genes were all compared to the corresponding genes in the reference genome, and the comparison rates were all over 98%, which further confirmed the reliability of the transcriptome data [18]. The enrichment pathway map showed the top 20 pathways with the most reliable enrichment significance (Fig. 5). Phenylpropanoid biosynthesis is the major pathway for flavonoid synthesis, and DEGs were enriched to the phenylpropanoid biosynthesis pathway in T1_K vs. T1_P. Proteins related to the flavonoid synthesis pathway were enriched in T2_ K vs. T2_P and T3_K vs. T3_P. And based on the KEGG enrichment maps of the same tissues at different growth stages, it could be seen that the flavonoid synthesis pathway was significantly enriched in both kernels and peels of the comparative groups, except for the T2 vs. T3, which was consistent with the trend of differences in flavonoid metabolites among different groups (Fig. S6, Fig. S7, Table S3).

### Hypothesized flavonoid biosynthesis pathways in gardenia fruits

**Fig. 6:**
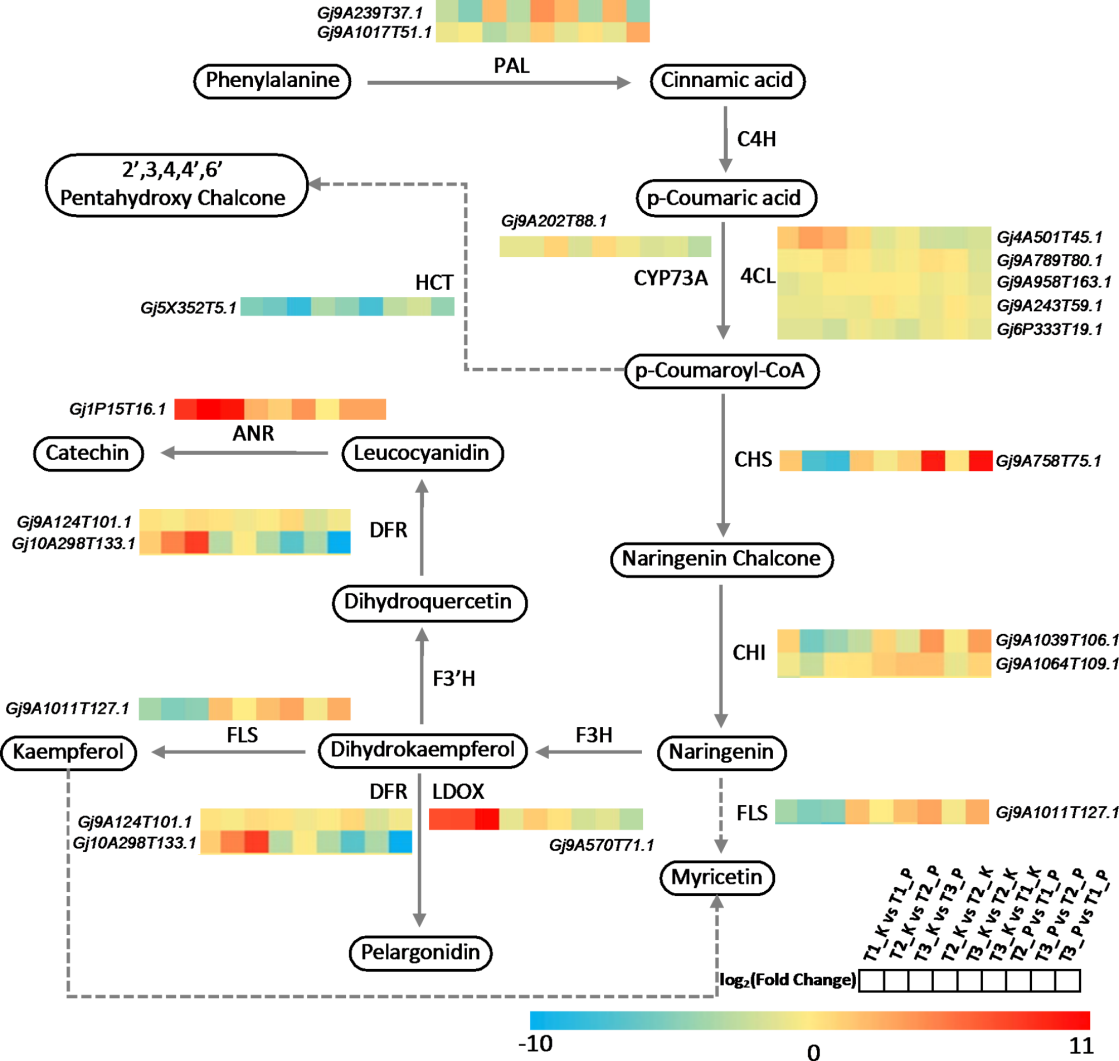
**Gene expression profiles of hypothetical flavonoid synthesis pathways and key enzymes in Gardenia fruits**. PAL, Phenylalanine ammonia-lyase; C4H, Cinnamate 4-hydroxylase; CYP73A, Cinnamate 4-hydroxylase; 4CL, 4-coumaroyl-CoA ligase; CHS, Chalcone synthase; CHI, Chalcone isomerase; FLS, Flavonol synthase; DFR, Dihydroflavonol reductase; F3H, Flavanone 3-hydroxylase; F3’H, Flavonoid 3’-hydroxylase; LDOX, Leucoanthocyanidin dioxygenase; HCT, Hydroxycinnamoyl transferase; ANR, Anthocyanidin reductase ((2 S)-flavan-3-ol-forming)

Pathway annotation analysis of DEGs helps to further understand the function of genes. Combined with the KEGG pathway annotation, we compared the DEGs enriched in the flavonoid synthesis pathway. We combined the results of differential metabolites and DEGs during flavonoid biosynthesis with the aim of more intuitively understanding the relationship between metabolites and genes during flavonoid biosynthesis (Fig. 6). As could be seen from Fig. 6, the expression of genes related to flavonoid synthesis varied significantly in different growth stages and different tissues of gardenia fruits. For example, the expression of chalcone synthase Gj9A758T75.1 was significantly down-regulated in the kernel compared with the peel at the same growth stage; the expression of the gene was not significantly different in the kernel but significantly up-regulated in the peel at different growth stages.

In addition, comparing the expression of dihydroflavonol reductase Gj10A298T133.1 among different samples, the expression of the gene was significantly up-regulated in the kernel compared to the peel at the same growth stage; the expression of the gene was significantly down-regulated in both the kernel and the peel at different growth stages. The number of genes encoding enzymes essential for flavonoid biosynthesis was abundant, and their expression demonstrated substantial variation during different stages (Table S8). Notably, the expression trends of these genes did not show a consistent correlation with metabolites, which may reflect the complexity of the gene-to-protein expression process, in which transcription factor deficiencies may be a factor [19].

### Validation of RNA-Seq data

**Fig. 7:**
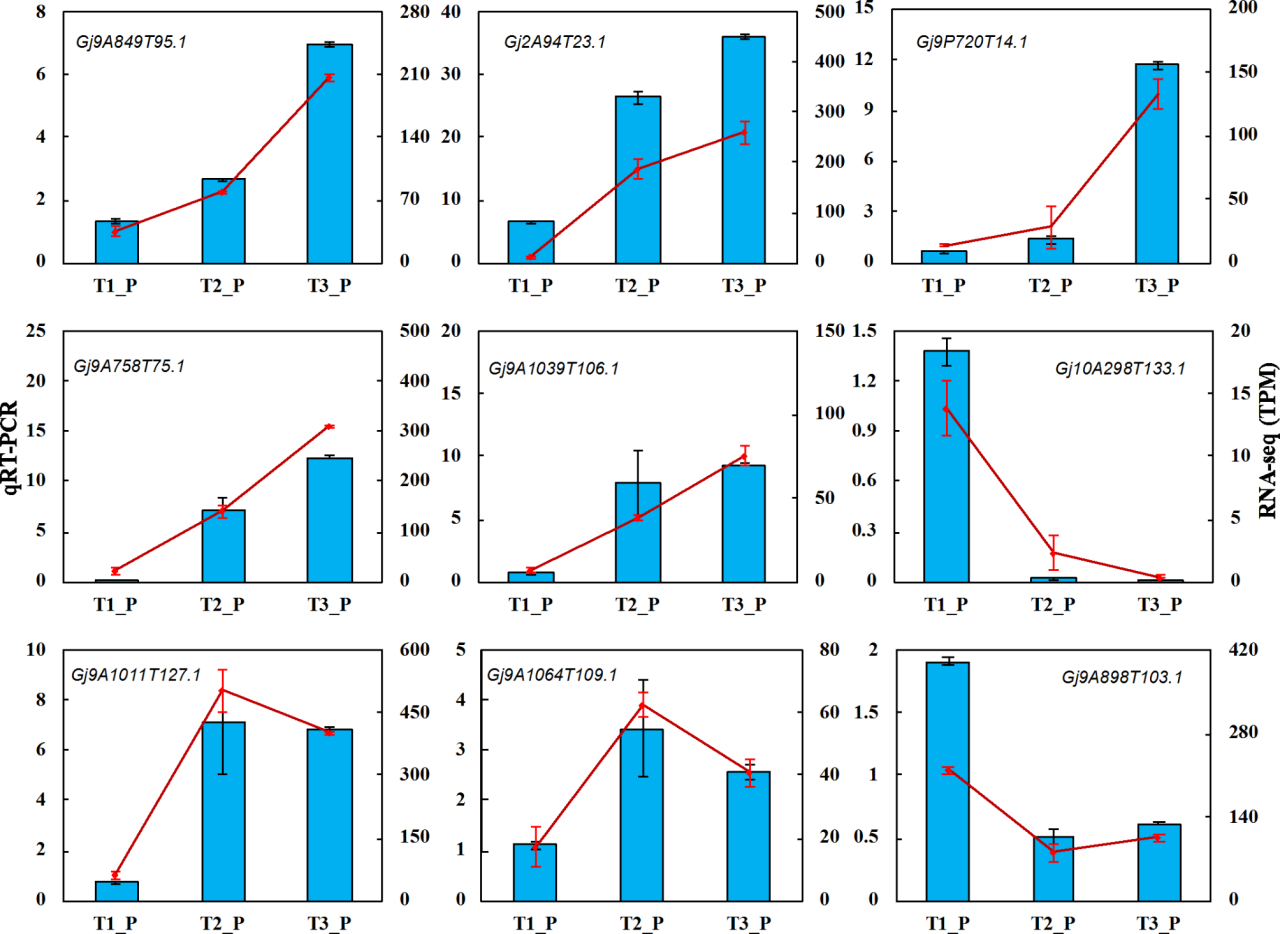
**QRT-PCR was performed to validate 9 DEGs identified by RNA-seq in gardenia peel at different growth stages**. Red lines indicate qRT-PCR results. Histogram indicates the RNA-seq results. T1 represent green ripe fruits (sampled on July 16), T2 represent semi-ripe fruits (sampled on August 15) and T3 represent yellow ripe fruits (sampled on October 20). Peel (P), kernel (K)

Nine DEGs were randomly selected for qRT-PCR analysis (Fig. 7), which confirmed the confidence of the RNA-Seq data (Table S9).

## Discussion

Flavonoids are a group of plant polyphenol secondary metabolites that are essential for plant color formation [20]. When plants were subjected to stress, flavonoids also acted as a signaling molecule to activate defense-related signaling pathways and regulatory mechanisms, thereby enhancing tolerance and resistance to adversity stress [21, 22]. The phenylpropanoid pathway is the first three steps in the flavonoid synthesis pathway [23], which converts phenylalanine to 4-coumaroyl-CoA via phenylalanine ammonia-lyase (PAL) and 4-coumaroyl-CoA ligase (4CL) [24], and then converts 4-coumaroyl-CoA to flavonoids sequentially via chalcone synthase (CHS), chalcone isomerase (CHI), flavonol synthase (FLS), dihydroflavonol reductase (DFR), and anthocyanin reductase (ANR) [25–30]. Flavonoids are a very important class of metabolic components in gardenia fruit, which play an important role in the color of the fruit, antioxidant, and other aspects. Geniposides is another important constituent in gardenia, and its medicinal value in gardenia fruit also has nonnegligible role. The abundant presence of flavonoids and geniposides is thought to account for the advantageous properties associated with gardenia fruits [31]. Therefore, analyzing the metabolites of gardenia fruits at different developmental stages is not only beneficial to understand the coloring process of gardenia fruits, but also beneficial to the development of gardenia fruits health products.

In this study, we evaluated the metabolites of three typical developmental stages of the peel and kernel of gardenia using UPLC-MS/MS and explored the changes of related compounds and their respective genes at different developmental stages by transcriptome analysis. The results showed that there were significant differences in the contents of flavonoids and different expression of flavonoid biosynthetic genes at different developmental stages and in different tissues, which were consistent with the results on mulberry leaf, blueberries, Kiwifruits and sugarcane species, indicating that the metabolism of flavonoids was genetically regulated at different developmental stages and among different tissues, and their anabolic pathways are highly conserved in plants [32–35]. And the metabolite species were not consistent with previous studies, which may be due to the different species of gardenia, suggesting that the genotype plays a decisive role in its metabolite species [36]. According to the above data analysis shows, in the gardenia fruit flavonoids active ingredient extraction research process, determine the best research period is very necessary and meaningful. This study on the whole growth and development stage of flavonoids active substance change rule of the pre-mapping, the research results clear the gardenia fruit different growth period biological active component difference and dig the related gene, which for the follow-up study of the gardenia flavonoids active substance accumulation law and has important significance.

However, for the exploration of the geniposides pathway, we did not find sufficient evidence for the expression patterns of the key genes involved. It has been suggested that geniposides were derived from terpenoids and synthesized from the upstream mevalonate (MVA) pathway and the 2-C-methyl-d-erythritol-4-phosphate (MEP) pathway [37], which is not consistent with our study. This may be due to the complex role of geniposides in the metabolic pathway, which involves the regulation of multiple metabolic components and reaction pathways. Although this study failed to further confirm the metabolic pathway and regulatory mechanism, we believed that the study of this gene pathway still deserves to be explored in depth in future studies. Therefore, we will further explore the association of geniposides with other metabolic pathways in future studies to better elucidate the regulatory mechanisms of geniposides and other metabolic components in gardenia fruits.

## Conclusion

In summary, this study comprehensively analyzed the transcriptome and metabolome of gardenia fruit, revealed the regulatory network of flavonoid synthesis in gardenia fruit, identified key regulatory genes, and discovered the key links in the metabolic pathway. This provided an important reference for further in-depth study on the formation mechanism of potent components in gardenia. At the same time, we found that the flavonoid metabolic pathway of gardenia fruit had a certain spatiotemporal pattern and changed during fruit development and maturation. Therefore, the results of this study provided a theoretical basis and experimental basis for improving the quality of gardenia herbs and developing related products.

## Materials and methods

### Plant materials

The gardenia variety used for the experiment was “Linhai No.1”, a Chinese native variety jointly bred by Hunan Hi-Tech Bio-Agro Co.,Ltd and Chinese academy of forestry, grown in Changsha, Hunan Province, China, with normal field management, and samples of gardenia fruits were sampled at three different growth stages on July 16, August 15, and October 20, 2019, with six fruits were collected at each stage as a replicate sample. The peel (P) and kernel (K) were cleaned and placed in liquid nitrogen, then stored in -80℃ refrigerator until assayed.

### Sample preparation and extraction for metabolomic analysis

We freeze-dried peels and kernels of gardenia fruits at different stages of growth (Scientz-100 F, Ningbo, China) and ground them for 1.5 min at 30 Hz in a grinder (MM 400, Retsch). The 100 mg of powder was weighed and dissolved in 1.2 ml of 70% methanol extraction solution (v/v) and vortexed for 30 s every 30 min for 6 times to improve the extraction rate, and then left overnight at 4 °C. Incubation was for overnight, then centrifugation at 12,000 g for 10 min, followed by filtering the supernatant through a microporous membrane (0.22 μm; ANPEL, Shanghai, China). Filtrates were then stored in injection vials for UPLC-MS/MS analysis. As a final step, all samples were mixed in equal amounts to ensure that the analytical conditions were stable.

### UPLC and ESI-Q TRAP-MS/MS conditions

Data from gardenia fruit samples were collected using UPLC-ESI-MS/MS (Ultra Performance Liquid Chromatography-Electrospray Ionization-Tandem Mass Spectrometry) (Shim-pack UFLC SHIMADZU CBM30A system, MS/MS, Applied Biosystems 6500 QTRAP). In relation to the instrument analytical parameters, the column used was an ACQUITY UPLC HSS T3 C18 (1.8 μm, 2.1 mm * 100 mm, Waters). Mobile phase A comprised of distilled water containing 0.04% acetic acid (v/v), while mobile phase B consisted of acetonitrile with 0.04% acetic acid (v/v). A temperature of 40 °C was set for the column, a flow rate of 0.35 mL/min was used, and the injection volume was 4 µL, and the elution gradient program was as shown in Table 1: specifically, 95% A, 5% B at the starting condition, at the 9 min a linear gradient to 5% A, 95% B concentration was maintained for 1 min, followed by adjustment to 95% A and 5.0% B was maintained for 3 min.

**Table 1:** Metabolites gradient elution procedure

Time (min)	A %	B %
0	95	5
9	5	95
10	95	5
13	95	5

Subsequently, the measured solutions were connected to ESI-triple quadrupole-linear ion trap (Q TRAP) -MS. ESI source parameters were: ion source, turbo spray; source temperature 550◦ C; ion injection voltage (IS) 5500 V (positive)/ 4500 V (negative); ion source gas I (GSI), gas II (GSII), and curtain gas (CUR) were set to 50, 60, and 25.0 psi with a high collision gas (CAD), respectively. QQQ scans were obtained in MRM experiments with collisional gas (nitrogen) as the medium. Further DP and CE optimizations were performed for individual MRM transitions. Referring to the method of A-L Liu, Y-H Wang, T-Y Wang, Y Zhu, P Wu and L-J Li [38] with slight modifications.

### Qualitative and quantitative analysis of metabolites

The data were collected using the software Analyst 1.6.1, based on the Metabolite Information Public Database and the Metabolome Platform Reference Material Database, and the substances were characterized based on secondary spectral information. In this analysis, isotopic signals, duplicate signals containing K^+^, Na^+^, and NH4^+^, and fragment signals that are themselves other larger molecular weight substances, were removed [39]. Multiple reaction monitoring (MRM) modes of triple quadrupole mass spectrometry were utilized for quantitative analysis [40].

### Differential metabolite screening

The orthogonal partial least squares discriminant analysis (OPLS-DA) mode 1st principal component variable importance value projection value (VIP ≥ 1), differential metabolites were screened using the method of difference multiplier value (FC ≥ 2 or FC ≤ 0.5) and *P*-value of t-test (*P*-value ≤ 0.5).

### Transcriptome sequencing and data analysis

The extraction of total RNA from gardenia fruit samples was carried out using TRIzol reagent (Invitrogen, CA, USA) following the manufacturer’s guidelines. Total RNA concentration, RIN value, and 28S/18S ratio were measured using an Agilent 2100 Bioanalter (Agilent RNA 6000 Nano Kit), and RNA purity was determined using a The RNA purity was measured using a NanoDropTM UV spectrophotometer. The cDNA library was constructed by enriching the mRNA containing the poly-A tail with Oligo dT beads, fragmenting the obtained RNA with interrupted buffer, reverse transcribed with random N6 primers, and synthesizing the second-strand cDNA with DNA polymerase I and RNase H. The end of the double-stranded DNA was leveled and the 5’ end was phosphorylated. The synthesized double-stranded DNA was ligated to the paired splice and enriched by PCR using specific primers. Following quality control, sequencing was conducted on the Illumina platform.

To obtain clean reads, we initially processed the raw reads using Cutadapt [41] and an internal Perl script to eliminate short-read-length and low-quality reads after quality control. Then FastQC (http://www.bioinformatics.babraham.ac.uk/projects/fastqc/) to verify the sequence quality. All downstream analyses are based on high-quality clean data. The de novo assembly of the transcriptome was performed with Trinity 2.4.0 [42] and the longest transcripts in the cluster were selected as Unigene.

All assembled Unigenes were compared using DIAMOND [43] with the Nr Protein Database (http://www.ncbi.nlm.nih.gov/), Gene Ontology (GO) (http://www.geneontology.org), SwissProt (http://www.expasy.ch/sprot/), Kyoto Encyclopedia of Genes and Genomes (KEGG) (http://www.genome.jp/kegg/) [44–46], and eggNOG (http://eggnogdb.embl.de/) databases for alignment with a threshold of EValue < 0.00001. The expression levels of Unigenes were performed by calculating TPM using Salmon [47, 48]. R package edgeR (Robinson et al., 2010) selected differentially expressed Unigenes with log_2_ (fold change) > 1 or log_2_ (fold change) < -1 and statistically significant (*P* value < 0.05) [49]. Finally, we mapped the assembled transcripts to the reference genome using BLAST software to ensure the accuracy of the data [18].

### Quantitative RT-PCR analyses

Nine differentially expressed genes were randomly selected, qRT-PCR was used to validate the sequences derived from sequencing results. Oligo 7 software was used to design primers (Table S9). Total RNA was extracted from gardenia fruits peel using FastPure Universal Plant Total RNA Isolation Kit (RC411-01, Vazyme, China), and then qRT- PCR was performed in QuantStud 6 (Thermo Scientific, USA) with reference to the method of S Gao, K Wang, N Li, Y Lv, B Cao, Z Chen and K Xu [50].

### Statistical analysis

In this experiment, all samples were randomly sampled, and each treatment contained 6 replicates. IBM SPSS statistical (SPSS26) was used to statistically analyze the experimental data from 6 independent biological replicates. One-way analysis of variance (ANOVA) was employed to perform statistical analysis on all data, followed by the application of Duncan’s test.

All methods in this paper have been implemented in accordance with the relevant guidelines/regulations/legislation.

### Electronic supplementary material

Below is the link to the electronic supplementary material.

Supplementary Material 1

Supplementary Material 2

Supplementary Material 3

## Data Availability

The sequence reads and expression data of the reported transcriptome have been deposited in the Large Data Center of the Beijing Institute of Genomics (BIG), Chinese Academy of Sciences (GSA: CRA011690).
