# Supplementary material for: Multi-omics analysis reveals the molecular basis of flavonoid accumulation in fructus of Gardenia (Gardenia jasminoides Ellis)
Source: BMC Genomics. 2023 Oct 4;24:588. doi: 10.1186/s12864-023-09666-x (PMC10548582; doi:10.1186/s12864-023-09666-x)
Supplement: Supplementary file 1 — Supplementary Material 1 [file 12864_2023_9666_MOESM1_ESM.docx]

**Additional files**

**Additional file 1: Fig. S1** Identified metabolites from level-one identification were classified into 24 and 25 HMDB super classes. (A) POS mode; (B) NEG mode. The x-axis represents HMDB super classes, and the y-axis represents the number of identified metabolites.

**Fig. S2** KEGG pathway classification: metabolites detected and annotated. (A) POS mode; (B) NEG mode. The x-axis represents level-2 terms of the KEGG pathway, and the y-axis represents the number of metabolites identified.

**Fig. S3** Identified metabolites classified into the top 20 KEGG pathways. (A) POS mode; (B) NEG mode. The x-axis represents the top 20 KEGG pathways and the y-axis represents number of identified metabolites involved in this pathway.

**Fig. S4.** Profile of two tissues and metabolites of gardenia fruit at different growth stages. (A) PCA analysis of metabolites of POS mode; (B) PCA analysis of metabolites of NEG mode. (C) Heatmap analysis of metabolites of different samples in NEG mode.

**Fig. S5.** Heatmap of differentially expressed genes among different tissues of Gardenia fruits at different growth stages. (A-E), T1_K vs T2_K vs T3_K, T1_P vs T2_P vs T3_P, T1_K vs T1_P, T2_K vs T2_P, T3_K vs T3_P. Red represents higher gene expression and blue represents lower gene expression.

**Fig. S6.** KEGG enrichment analysis of differentially expressed genes among different tissues of Gardenia fruits at different growth stages. (A-C), T2_K vs T1_K, T3_K vs T2_K, T3_K vs T1_K. Horizontal coordinates represent the enrichment factor, vertical coordinates represent the pathway definition, dot size represents the number of genes, and color represents the p-value.

**Fig. S7.** KEGG enrichment analysis of differentially expressed genes among different tissues of Gardenia fruits at different growth stages. (A-C), T2_P vs T1_P, T3_P vs T2_P, T3_P vs T1_P. Horizontal coordinates represent the enrichment factor, vertical coordinates represent the pathway definition, dot size represents the number of genes, and color represents the p-value.

**Additional file 2: Table S1** Statistics of identified metabolites

**Table S2** Statistics for differential metabolites between different group comparisons

**Table S3** Comparison of Flavonoid & Geniposide contents in fruit peel and kernel

**Table S4** Metabolite content of different treatments

**Table S5** Comparison of Flavonoid & Geniposide contents in fruit peel at different growth stages

**Table S6** Expression of Flavonoid & Geniposide-related genes

**Table S7** Expression of key genes in the Flavonoid pathway

**Table S8** Flavonoid pathway key gene expression heat map

**Table S9** The primer sequences of target and reference genes used in RT-qPCR analysis
